# Real world effectiveness of standard of care triple therapy versus two-drug combinations for treatment of people living with HIV

**DOI:** 10.1371/journal.pone.0249515

**Published:** 2021-04-08

**Authors:** Ramón Teira, Helena Diaz-Cuervo, Filipa Aragão, Sophie Marguet, Belén de la Fuente, Maria Jose Muñoz, Nadia Abdulghani, Esteban Ribera, Pere Domingo, Elisabeth Deig, Joaquim Peraire, Bernardino Roca, Marta Montero, Maria José Galindo, Alberto Romero, Nuria Espinosa, Fernando Lozano, María Dolores Merino, Elisa Martínez, Paloma Geijo, Vicente Estrada, Josefina García, M. Antonia Sepúlveda, Juan Berenguer

**Affiliations:** 1 Hospital de Sierrallana, Torrelavega, Spain; 2 Gilead Sciences, Medical Affairs, Stockley Park HEOR, Spain; 3 Maple Health Group, New York, New York, United States of America; 4 NOVA National School of Public Health, Public Health Research Centre, Universidade NOVA de Lisboa, Lisboa, Portugal; 5 Amaris Consulting, Health Economics and Market Access (HEMA), Levallois-Perret, France; 6 Hospital de Cabueñes, Gijón, Spain; 7 Hospital de Basurto, Bilbao, Spain; 8 Hospital Arnau de Vilanova, Lleida, Spain; 9 Hospital Vall d´Hebrón, Barcelona, Spain; 10 Hospital Santa Creu i Sant Pau, Barcelona, Spain; 11 Hospital General, Granollers, Spain; 12 Hospital Joan XXIII, Tarragona, Spain; 13 Hospital General, Castellón, Spain; 14 Hospital La Fé, Valencia, Spain; 15 Hospital Clínico, Valencia, Spain; 16 Hospital Universitario de Puerto Real, Puerto Real, Spain; 17 Hospital Virgen del Rocío, Sevilla, Spain; 18 Hospital de Valme, Sevilla, Spain; 19 Hospital Infanta Elena, Huelva, Spain; 20 Complejo Hospitalario de Albacete, Albacete, Spain; 21 Hospital Virgen de la Luz, Cuenca, Spain; 22 Hospital Clínico de San Carlos, Madrid, Spain; 23 Hospital Santa Lucía, Cartagena, Spain; 24 Hospital Virgen de la Salud, Toledo, Spain; 25 Hospital General Universitario Gregorio Marañón, Madrid, Spain; University of Colorado Denver Skaggs School of Pharmacy and Pharmaceutical Sciences, UNITED STATES

## Abstract

**Background:**

Since 1996, the standard of care (SOC) therapy for HIV treatment has consisted of a backbone of two nucleoside analogue reverse transcriptase inhibitors (NRTI) paired with a third agent. Use of two-drug combinations (2DC) has been considered for selected patients to avoid toxicities associated with the use of NRTIs. This study aimed to compare the real-world outcomes of integrase strand transfer inhibitor (INSTI)-containing triple therapy (TT) to dolutegravir- (DTG) and/or boosted protease inhibitor (bPI)-based 2DC in a large Spanish cohort of HIV patients.

**Methods:**

A retrospective analysis was performed using data from the VACH cohort, a prospective multicentre Spanish cohort of adult HIV patients. All treatment experienced patients initiating a TT of an INSTI combined with two NRTIs or a 2DC-containing DTG and/or a bPI between 01/01/2012 and 01/06/2017 were included. The unit of analysis was patient-regimens. The overall sample analysis was complemented with two sub-analyses. The first sub-analysis focused on patients treated with a backbone plus DTG compared to those treated with DTG+ one other antiretroviral. The second sub-analysis focused on patients with HIV RNA<50 copies/mL at baseline, irrespective of the regimen used. The following endpoints were assessed: time to discontinuation for any reason, time to switch due to virologic failure, and time to switch due to toxicity (reasons for discontinuation according to clinician report in the database). Time-to-event analyses were conducted using Kaplan–Meier survival curves and Cox regression models.

**Results:**

Overall 7,481 patients were included in the analysis, contributing to 9,243 patient-regimens. Patient characteristics at baseline differed among groups, with the 2DC group being significantly older and having a higher proportion of women, a longer time on ART and a higher number of previous virologic failures. Median (95% Confidence Interval [C.I.]) time to switch was 2.5 years (2.3, 2.7) in 2DC group versus 2.9 years (2.7, 3.0) in TT. Adjusted hazard ratios (95% C.I.) for discontinuation due to any reason, virologic failure and toxicity in the 2DC vs TT group were 1.29 (1.15; 1.44), 2.06 (1.54; 2.77) and 1.18 (0.94; 1.48), respectively. Results were consistent in the two sub-analyses.

**Conclusion:**

In this analysis, time to discontinuation and probability of remaining free of virologic failure were significantly higher in patients on INSTI-based TT compared to DTG- and/or bPI-containing 2DC, with no differences in toxicity.

## Introduction

In 2016, there were 160,453 people newly diagnosed with HIV in 51 of the 53 countries in the World Health Organization (WHO) European Region, which corresponds to a rate of 18.2 newly diagnosed infections per 100,000 population [1]. Moreover, it is estimated that approximately 15% of those living with HIV in the European Union (EU) and European Economic Area (EEA) are not aware of their status [1].

Although the clinical, immunological, and virologic course of untreated HIV infection is variable, few untreated persons followed for more than 8–10 years remain without any evidence of disease progression [2]. The majority of the patients who do not receive antiretroviral therapy (ART) die within 2 years of the onset of acquired immunodeficiency syndrome (AIDS) [3].

Improvements in ART have transformed HIV from a terminal to a chronic disease. Patients who started ART in 2008–10 and are able to reach CD4+ T cell counts (CD4) of at least 350 cells/μL within one year after ART initiation have an estimated life expectancy approaching that of the general population [4].

ART itself has evolved over time from monotherapy to triple therapy (TT). The recommended combination regimen for treatment initiation consists of two nucleoside analogue reverse transcriptase inhibitors (NRTI) (“backbone”) paired with a third agent [5]. Since 1996, TT has been the standard of care therapy in HIV treatment.

Despite improvements in TT regimens, long-term toxicity of tenofovir disoproxil fumarate (TDF) and abacavir (ABC), led clinicians and researchers to look for solutions to help people living with HIV achieve the best possible long-term health. Exploratory strategies such as two-drug combinations (2DC) have been considered where neither TDF nor ABC are optimal options.

2DC strategies have been used in the past. With few exceptions, 2DC strategies have been associated with higher rates of virologic failure than TT in the clinical trial setting [5]. However, the SWORD 1&2 [6] randomized clinical trials showed that switching to dolutegravir (DTG) + rilpivirine (RPV) was non-inferior to remaining on TT in patients stably suppressed (viral load <50 copies per mL) for at least 6 months with no hepatitis B virus (HBV) coinfection, no history of virologic failure on a prior regimen, and no history of any resistance associated major protease inhibitors (PI), integrase strand transfer inhibitor (INSTI), NRTI, or non-nucleoside analogue reverse transcriptase inhibitors (NNRTI) mutation. In line with the SWORD 1&2 results, the TANGO randomized trial [7] demonstrated non-inferiority of switching to DTG/lamivudine (3TC) when compared to maintaining a tenofovir alafenamide (TAF)-containing regimen of at least three drugs, among patients suppressed for at least 6 months, without prior virologic failure, no historical NRTI or INSTI major resistance mutation, and no evidence of HBV infection. Lastly, the GEMINI 1&2 [8] randomized clinical trials showed that DTG+3TC was non-inferior to emtricitabine (F)/TDF+DTG in carefully selected treatment naïve patients with HIV-1 RNA ≤500,000 copies/mL, CD4 >200 cells/mL, no HBV coinfection, and no known resistance associated major PI, INSTI, NRTI, or NNRTI mutations.

One of the main concerns with 2DC is the potential for a lower resistance barrier, which may be more problematic with poorer adherence. Exclusion criteria for HIV treatment drug trials can be stringent and selection bias exists, making it difficult to extrapolate results into ’real-world’ settings [9]. Average adherence in the clinical trial setting is often at least 95% [10, 11] while in the real-world it is often below 80% [12] with a significant proportion of patients experiencing treatment gaps [13]. While older TT regimens required 95% adherence for optimal effectiveness [14–16], there is currently evidence to support that more contemporary TT regimens are more “forgiving” of suboptimal adherence; indeed, >80% adherence may be enough to maintain suppression [15–17].

Given the potential discrepancy between randomized clinical trials and effectiveness in real-world settings, this study aimed to evaluate the outcomes of INSTI-containing TT to DTG- and/or boosted PI (bPI)-containing 2DC in a large, Spanish, cohort of HIV patients.

## Materials and methods

### Data collection

The VACH cohort is a prospectively recruited Spanish cohort of 14,833 HIV-infected adult patients from 23 investigational centres across Spain. Enrollment started in 1997. Data are prospectively collected in an electronic case record form according to standardized criteria and are electronically stored in the *Aplicación de Control Hospitalario* (AC&H^™^). This application was specifically developed for the management of the cohort data: demographic data, HIV risk factors, Centers for Disease Control and Prevention stage according to 1993 definitions, HIV-1 treatment initiation date, the specific antiretroviral regimens used, the date of change of every drug and the reasons for change, CD4 cell count, plasma HIV RNA levels, blood cell counts and blood chemistry tests. Data are periodically recorded at each patient visit at intervals of approximately 3–4 months. All collected information is transformed into a standardized format and merged into a central dataset. The data passes an internal duplicate control at the central data centre that identifies patients by a unique code. Internal validation controls and quality controls of the data are used. Informed consent not required given the de-identified nature of the data. The study was reviewed and approved by Ethics Review Board of Cantabria IDIVAL.

### Patient selection

All patients in the VACH cohort switching between January 1^st^, 2012 and June 1^st^, 2017 were considered and checked for inclusion and exclusion criteria.

Only patients switching to the following regimens were considered:

- INSTI-containing TT: elvitegravir/cobicistat (E/C), raltegravir (RAL) or DTG combined with one of the following 2 NRTI backbones: emtricitabine (F)/TDF or 3TC/abacavir (ABC) or F/TAF.
- DTG or bPI–based 2DC: DTG or boosted (b) darunavir (DRV) or b-atazanavir (ATV) or b-lopinavir (LPV) combined with 3TC; DTG or bDRV combined with RPV; bDRV or bLPV combined with RAL; DTG plus bDRV

The unit of analysis was the patient-regimen, defined as a patient on a specific regimen. This meant that a given patient could contribute multiple times to the data if during the relevant period of analysis, they initiated a regimen of interest more than once. Patient-regimens were followed from date of regimen switch to discontinuation, loss-to-follow up, death or end of the observation period (June 30^th^, 2017), whichever happened first.

The following exclusion criteria were applied: (1) If an individual had participated in an interventional clinical trial while on a specific regimen, that patient-regimen information was excluded but the remaining patient-regimens for that individual during the study period would be included if eligible; (2) patient-regimens were excluded if reason of discontinuation of the previous regimen was: loss to follow-up, programmed interruption, intermittent treatment or intention of restoring wild type virus. Patient-regimens with data inconsistencies that could not be corrected were also excluded.

### Statistical analysis

Patient-regimens were stratified according to the type of ART regimen: TT versus 2DC. Baseline characteristics were compared using the Student’s t-test to compare two means and the *χ*^2^ test for categorical variables if all cells had expected counts more than 5; otherwise Fisher’s exact test was used.

The univariate and multivariate analyses performed targeted the following endpoints of interest: time to discontinuation for any reason, time to discontinuation due to virologic failure, time to discontinuation due to toxicity (classified in the database as due to adverse event, intolerance, avoiding long-term toxicity or drug interactions). Reasons for discontinuation were based on the clinicians’ report in the database.

Time-to-event analyses were conducted using Kaplan–Meier survival curves, comparing both groups via the log-rank test and the Cox proportional regression analysis. As potential confounders, the following variables were initially introduced in the Cox regression analysis: age, gender, AIDS diagnosis ever (Y/N: Yes/No), hepatitis C virus (HCV) (Y/N) co-infection, HBV (Y/N) co-infection, illicit drug use ever (Y/N), HIV RNA (copies/mL), CD4 counts (cells/mm^3^), duration on ART, number of previous ART regimens, number of previous virologic failures. All covariates were evaluated at patient-regimen initiation unless otherwise stated. Confounders with more than 20% of missing values were not included in the Cox model. A stepwise selection was used to select the covariates to be included in the final model using a p-value of 0.25 to enter the model and a p-value of 0.10 to remain in the model.

The analysis was performed on the overall sample as well as two pre-defined sub-analyses: (1) DTG-containing TT versus DTG-containing 2DC, and (2) all TT versus 2DC, among patients in the overall sample suppressed (defined as HIV RNA<50 copies/mL) at baseline.

All analyses were performed using SAS (Version 9.4, The SAS institute, Cary, NC).

## Results

### Overall sample

#### Antiretroviral regimen distribution and baseline characteristics

Overall, 7,481 patients were included in the analysis, contributing 9,243 patient-regimens. Out of the 7,481 distinct patients, 5,992 contributed to the TT group with 7,371 patient-regimens and 1,489 contributed to the 2DC group with 1,872 patient-regimens. 48 patient-regimens were excluded because of the reason for discontinuation of the previous regimen (92% discontinued due to intermittent treatment) and 19 because of unresolvable data inconsistencies. As shown in Table 1, DTG/+3TC/ABC (that is, in a single tablet or 2 pills) and E/C/F/TAF accounted for almost two thirds of the patient-regimens in TT; 60% of the 2DC regimens consisted of bDRV combined with either 3TC or RAL, and DTG+RPV (two pills given that at the time of the study no 2DC single tablet regimen was available).

**Table 1:** Antiretroviral therapy distribution, overall sample, by therapy group.

Triple Therapy (TT)		Two-Drug Combinations (2DC)	
ART Regimen	N (%)	ART Regimen	N (%)
DTG/+3TC/ABC*	2686 (36.4%)	bDRV + 3TC	548 (29.3%)
E/C/F/TAF	1883 (25.6%)	DTG + RPV	293 (15.7%)
E/C/F/TDF	1083 (14.7%)	bDRV + RAL	292 (15.6%)
F/TDF+RAL	739 (10.0%)	DTG + bDRV	249 (13.3%)
3TC/ABC+RAL	576 (7.8%)	bDRV + RPV	207 (11.1%)
F/TDF+DTG	404 (5.5%)	DTG + 3TC	146 (7.8%)
		bLPV + 3TC	95 (5.1%)
		bLPV + RAL	42 (2.2%)

ART: antiretroviral therapy; 3TC: lamivudine; ABC: abacavir; DTG: dolutegravir; F: emtricitabine; TAF: tenofovir alafenamide; TDF: tenofovir disoproxil fumarate; E/C: elvitegravir/cobicistat; b: booster; RAL: raltegravir; bDRV: boosted darunavir; RPV: rilpivirine; bLPV: boosted lopinavir.

*While most patient-regimens were on DTG/3TC/ABC (one tablet) some were on two tablets (3TC/ABC) + DTG. For the purpose of the present analysis, no distinction was made between the two and DTG/+3TC/ABC is used as notation for both cases.

Statistically significant differences were observed between the two groups as detailed in Table 2. At baseline, the 2DC group was older (mean age 50.0 vs 47.4, p<0.0001) and included a higher proportion of women (27.8% vs 23.1%, p<0.0001). The 2DC group had a higher proportion of patients co-infected with HCV (47.2% vs. 39.4%, p<0.0001) and more patients with history of illicit drug use (40.3% vs. 32.9%, p<0.0001) compared to the TT group. Patients on 2DC had been on ART for 3 years longer on average than the TT group (14.6 vs. 11.6, p<0.0001), were more likely to have had a previous AIDS diagnosis (32.3% vs. 24.9%, p<0.0001) and had a higher number of previous virologic failures (2.3 vs. 1.0, p<0.0001).

**Table 2:** Sociodemographic and clinical characteristics at baseline, overall sample.

Characteristics at Baseline	Triple Therapy (N = 5,992 patients, 7,371 patient-regimens)	Two-Drug Combinations (N = 1,489 patients, 1,872 patient-regimens)	P-value*
Age, Mean (SD)	47.4 (10.1)	50.0 (9.2)	**< .0001**
Male	76.9%	72.2%	**< .0001**
Country of Origin, %			**0.008**
Spain	72.8%	75.8%	
Other	27.2%	24.2%	
HIV Transmission Risk Group			**< .0001**
Heterosexual	29.1%	33.2%	
Homosexual or Bisexual	35.0%	23.9%	
People Who Inject Drugs	33.7%	40.9%	
People Who Inject Drugs and Homosexual	1.3%	1.2%	
Other	1.0%	0.8%	
Age at Diagnosis, Mean (SD)	33.3 (10.2)	32.6 (10.4)	**0.017**
Prior AIDS Diagnostic, Yes	24.9%	32.3%	**< .0001**
Nadir CD4+ T cell count			**< .0001**
<50 cells/μL	17.1%	24.1%	
51–200 cells/μL	32.7%	36.8%	
201–350 cells/μL	27.2%	25.0%	
≥350 cells/μL	23.0%	14.1%	
CD4+ T cell count <350 cells/μL	23.4%	22.8%	0.618
HIV RNA <50 copies/mL	76.9%	74.6%	**0.034**
Illicit drug use, Yes	32.9%	40.3%	**< .0001**
Hepatitis C virus coinfection, Yes	39.4%	47.2%	**< .0001**
Hepatitis B virus coinfection, Yes	4.7%	1.6%	**< .0001**
Number of years on ART, Mean (SD)	11.6 (7.5)	14.6 (7.2)	**< .0001**
Number of previous ART regimens, Mean (SD)	4.4 (3.6)	7.0 (5.3)	**< .0001**
Number of virologic failures, Mean (SD)	1.0 (1.8)	2.3 (3.7)	**< .0001**

SD: standard deviation; HIV: Human immunodeficiency virus; AIDS: acquired immunodeficiency syndrome; RNA: ribonucleic acid; ART: antiretroviral therapy; RNA; ribonucleic acid.

#### End of follow-up status, overall sample

Overall, the TT group contributed 8,307 patient-years of follow-up while the 2DC group contributed 2,154 patient-years. By the end of follow-up period, a higher proportion of patients had switched treatment in the 2DC group (31.4% vs. 24.2%) with a primary contributor being switch due to virologic failure. A significantly higher number of 2DC patient-regimens discontinued due to virologic failure compared to TT (14.0% vs 6.7%, p<0.0001) (Table 3). There was no significant difference in discontinuations due to adverse events. A higher proportion of patients discontinued in the TT group to avoid long-term toxicities (4.5% vs 1.2%, p = 0.0002). It is worth noting that the proportion lost to follow-up was identical (8.6%) in both groups in this real-world setting which is one important marker of similarity between the two cohorts.

**Table 3:** Status at end of observation period, overall sample, by treatment group.

End of the Observation Period Status, N (%)	Triple Therapy (TT)	Two-Drug Combinations (2DC)	P-value*
Remained on treatment	4919 (66.7%)	1110 (59.3%)	
Change of treatment	1779 (24.2%)	587 (31.4%)	
Due to Virologic failure	**119 (6.7%)**	**82 (14.0%)**	**< .0001**
Due to Adverse event	372 (20.9%)	104 (17.7%)	0.094
Due to Intolerance	11 (0.6%)	4 (0.7%)	0.773
To Avoid Long-Term Toxicities	**80 (4.5%)**	**7 (1.2%)**	**0.0002**
Due to Simplification	320 (18.0%)	98 (16.7%)	0.477
Due to Drug Interactions	79 (4.4%)	21 (3.6%)	0.368
Due to Lost to Follow-up	18 (1.0%)	3 (0.5%)	0.320
Due to Others	764 (43.0%)	264 (45.0%)	0.389
*Missing value*	*16*	*4*	
Lost to follow-up	637 (8.6%)	161 (8.6%)	
Death	36 (0.5%)	14 (0.8%)	

#### Survival analysis, overall sample

**Fig 1:**
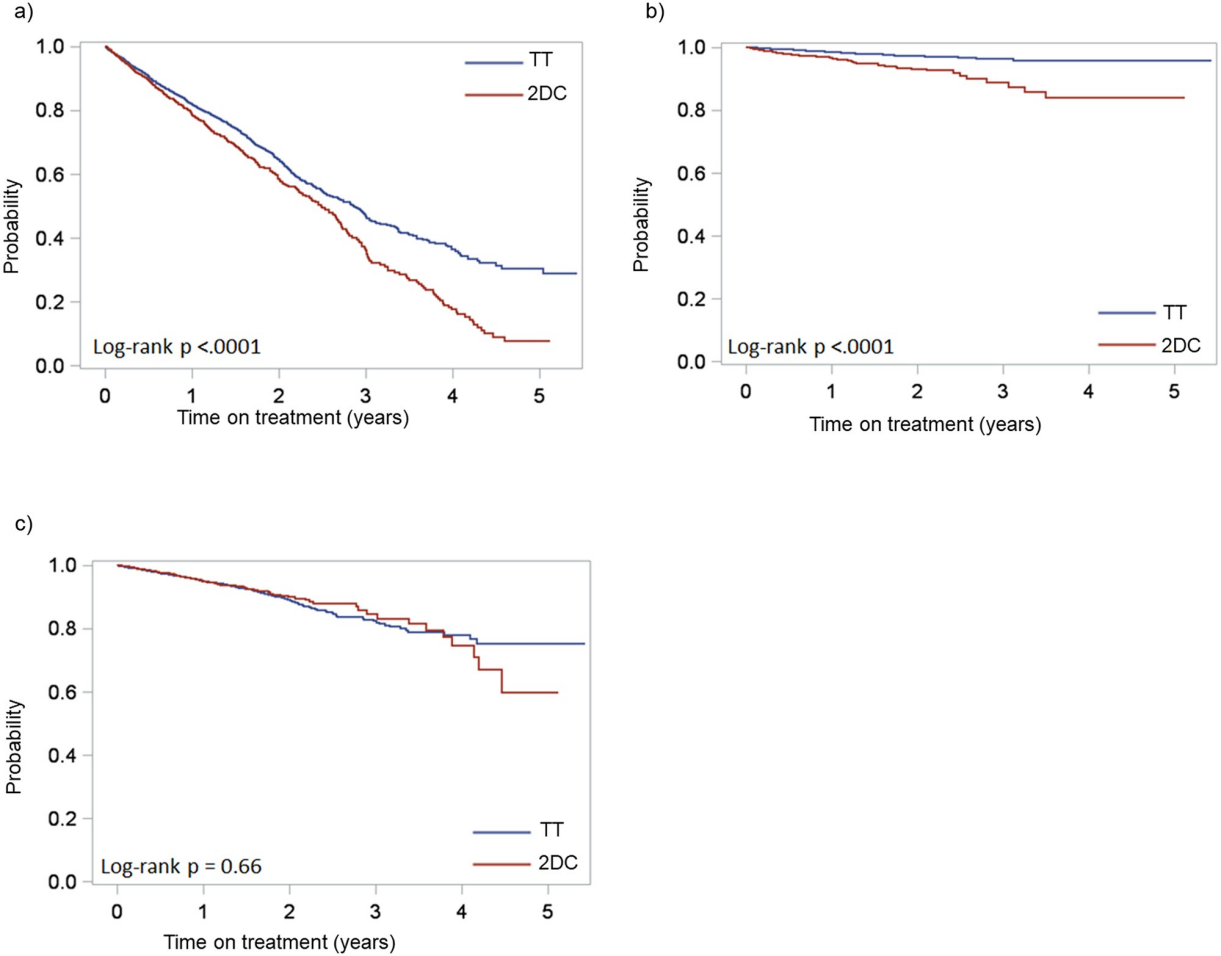
Kaplan-Meier curve for two-drug combinations versus triple therapy: a) time to discontinuation), b) switch due to virologic failure, c) switch due to toxicity. TT: triple therapy; 2DC: two-drug combination.

Fig 1 presents the Kaplan-Meier unadjusted analysis for time to discontinuation, stratified by reason for discontinuation. Longer time to discontinuation for any reason was observed in the TT group, where the probability of persistence at 5 years was 30.3% compared to 7.7% in the 2DC. Median time to discontinuation for any reason was 0.4 years longer in the TT group (2.9 vs. 2.5 years, p<0.0001) (Fig 1a). When exploring reasons for and time to discontinuation, there was a statistically significant difference in discontinuations due to virologic failure in favor of the TT group (p<0.0001) (Fig 1b). There was no statistically significant difference in discontinuation due to toxicity (p = 0.66), as shown in Fig 1c.

As previously described, some baseline characteristics differed among groups. It was thus important to account for potential confounders in multivariate regression. Table 4 shows the adjusted hazard ratios (aHR) obtained from the final model of the Cox Proportional hazard regression. Consistent with results of the Kaplan-Meier analysis, the risk of discontinuation for any reason was 29% (95% C.I.: [15%, 44%], p<0.0001) higher in the 2DC group compared to the TT group. The risk of discontinuation for virologic failure was also significantly higher in the 2DC group (aHR: 2.06, 95% C.I. [1.54, 2.77], p<0.0001). Differences in switches due to toxicity were not statistically significant (aHR: 1.18 [0.94, 1.48], p = 0.16).

**Table 4:** Final Cox model for two-drug combinations versus triple therapy time to discontinuation, switch due to virologic failure and switch due to toxicity, overall sample.

	Hazard Ratio [95% Confidence Interval], P-value		
Characteristics at initiation of the patient-regimen	Time to Discontinuation	Switch Due to Virologic Failure	Switch Due to Toxicity
Therapy group (2DC vs TT)	**1.29 [1.15, 1.44], p<0.0001**	**2.06 [1.54, 2.77], p<0.0001**	1.18 [0.94, 1.48], p = 0.16
Age, per year older	**1.00 [1.00, 1.01], p = 0.09**		**1.01 [1.00, 1.02], p = 0.003**
Hepatitis B virus coinfection, (Y/N)	**1.31 [1.06, 1.62], p = 0.013**		1.39 [0.93, 2.06], p = 0.11
Illicit drug use, (Y/N)	**1.33 [1.20, 1.47], p<0.0001**	**1.40 [1.05, 1.85], p = 0.021**	
Male (Y/N)	**0.88 [0.79, 0.97], p = 0.013**		
HIV RNA (≥50 vs. < 50)	**1.44 [1.30, 1.59], p<0.0001**	**4.68 [3.51, 6.23], p<0.0001**	
Years on antiretroviral therapy	**0.96 [0.95, 0.97], p<0.0001**		**0.89 [0.88, 0.91], p<0.0001**
Number of previous regimens	**1.06 [1.04, 1.07], p<0.0001**	**1.07 [1.06, 1.09], p<0.0001**	**1.12 [1.08, 1.17], p<0.0001**
Number of previous virologic failures			**0.92 [0.86, 0.97], p = 0.0039**

Y/N: Yes/No; 2DC: two-drug combination; TT: standard of care triple therapy; HIV: Human Immunodeficiency Virus; RNA: ribonucleic acid.

Note: Grey boxes reflect variables that were dropped in stepwise regression considering a 0.2 cut-off.

### Sub-samples: (1) dolutegravir-containing triple therapy versus dolutegravir-containing two-drug combinations and (2) HIV RNA <50 copies/mL at switch

#### Antiretroviral therapy and patients´ baseline characteristics

In the DTG-containing sub-analysis, all patient-regimens including DTG were included. Patient-regimens were then divided in two groups: DTG-containing TT (N = 3,090) and DTG-containing 2DC (N = 688). The distribution by antiretroviral combination is described in Table 5. Almost 90% were on DTG/+3TC/ABC in the DTG-containing TT group, and DTG + RPV was the most frequent combination (42.6%) in the DTG-containing 2DC group.

**Table 5:** Antiretroviral therapy distribution within dolutegravir containing therapy sub-sample, by treatment group.

Antiretroviral Therapy	Percentage
**Triple Therapy (n = 3,090)**	
DTG /+ 3TC /ABC	86.9%
F/TDF + DTG	13.1%
**Two-Drug Combinations (n = 688)**	
DTG + RPV	42.6%
DTG + bDRV	36.2%
DTG + 3TC	21.2%

DTG: dolutegravir; 3TC: lamivudine; ABC: abacavir; F: emtricitabine; TDF: tenofovir disoproxil fumarate; RPV: rilpivirine; bDRV: boosted darunavir.

The subset of suppressive patient-regimens was defined as those with HIV RNA <50 copies/mL at switch. Among suppressed at switch patients, the distribution of ART combinations was similar to that of the overall sample (Tables 5 and 6). A total of 6,982 patient-regimens were included in this sub-analysis, with 20% of those contributing to the 2DC group.

**Table 6:** Antiretroviral therapy distribution within suppressed at baseline sub-sample, by treatment group.

Antiretroviral Therapy	Percentage
**Triple Therapy (n = 5,596)**	
DTG /+ 3TC/ABC	37.0%
E/C/F/TAF	27.7%
E/C/F/TDF	13.3%
F/TDF+RAL	9.2%
DTG/3TC+RAL	8.2%
F/TDF+DTG	4.6%
**Two-Drug Combinations (n = 1,386)**	
bDRV + 3TC	31.6%
DTG + RPV	16.9%
bDRV + RAL	13.9%
bDRV + RPV	10.9%
DTG + 3TC	9.9%
DTG + bDRV	9.4%
bLPV + 3TC	5.9%
bLPV + RAL	1.5%

DTG: dolutegravir; 3TC: lamivudine; ABC: abacavir; E/C: elvitegravir/cobicistat, F: emtricitabine; TAF: tenofovir alafenamide; TDF: tenofovir disoproxil fumarate; RAL: raltegravir; bDRV: boosted darunavir; RPV: rilpivirine; bLPV: boosted lopinavir.

A detailed description of baseline sociodemographic and clinical characteristics for each of the sub-analyses is provided in the S1 Table. In essence, the pattern in each of the subsamples was similar to that of the overall sample.

In the DTG-containing subsample, patients in the 2DC group were older than those on TT (mean age: 50.6 vs. 48.9 years, p<0.0001), and there was a higher proportion of women in the 2DC group compared to the TT group (32.4% vs. 23.4%, p<0.0001). A higher proportion of patients with prior AIDS diagnoses was also observed in the 2DC group (32.1% vs. 25.8%, p = 0.0008). The 2DC group was also more experienced than the TT group, with an average number of prior regimens of 7.8 vs. 4.6 (p<0.0001). In contrast to the overall sample and the suppressed at switch sub-group, differences in HCV coinfection and illicit drug use were not statistically significant (HCV: 42.7% vs. 39.8%, p = 0.187; illicit drug use: 35.0% vs. 32.7%, p = 0.244).

In the suppressed at baseline subsample, patients in the 2DC group were older than those on TT (mean age: 50.7 years-old vs. 48.0 years-old, p<0.0001). There was a higher proportion of women in the 2DC group compared to TT group (27.2% vs. 22.9%, p = 0.001) and a higher proportion of patients with prior AIDS diagnoses (29.9% vs. 23.6%, p<0.0001). The 2DC group was also more treatment-experienced than the TT group, with an average number of prior regimens of 7.0 vs. 4.4 (p<0.0001). The proportion of patients with HCV diagnosis was higher in the 2DC group than in the TT group (39.0% vs. 47.5%, p<0.0001), as was the proportion of patients with history of illicit drug use (32.6% vs. 40.8%, p<0.0001)

#### End of follow-up status

At the end of the observation period, consistent with the overall sample analysis, more patients on TT persisted on the regimen compared to patients on 2DC. In the DTG-containing sub-analysis 80.4% of the TT group versus 71.8% of the 2DC group remained on the regimen. In the suppressed at baseline sub-analysis, 69.9% of the TT group versus 63.3% of the 2DC group remained on the regimen.

A lower proportion of virologic failures occurred in the TT group compared to the 2DC group (DTG-containing sub-analysis: 8.2% vs. 18.7%; HIV RNA<50 copies/mL at baseline sub-analysis: 3.8% vs. 8.1%).

In the DTG-containing sub-analysis, the proportion of patients discontinuing treatment due to adverse events was higher in the TT group (21.8% vs 11.7%, p = 0.004), but there was no difference between groups in the suppressed patient sub-analysis (21.9% vs. 20.4%, p = 0.542).

Switches to avoid long-term toxicity did not differ in the DTG-containing sub-analysis (p = 0.122) but occurred more frequently in the TT group when considering patients with HIV RNA<50 copies/mL at baseline (5.4% vs. 1.2%, p<0.001), (S2 Table).

#### Time to event

**Fig 2:**
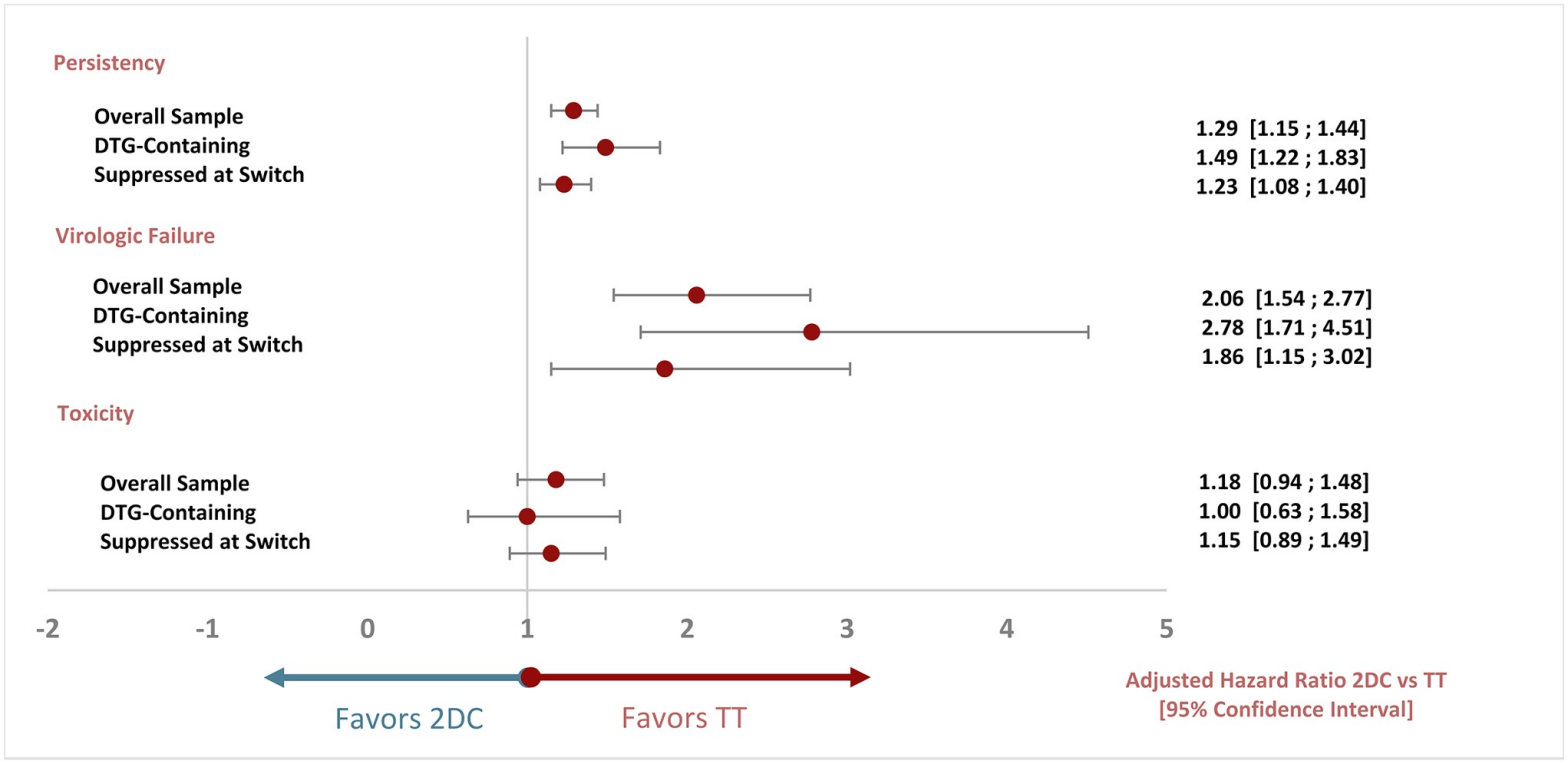
Adjusted hazard ratio for two-drug combinations versus triple therapy forest plot, by sample and endpoint. DTG: dolutegravir; 2DC: 2 drug combinations; TT: triple therapy.

Results in both sub-analyses were consistent with those of the overall sample. Fig 2 summarizes aHR for 2DC vs TT by reason for discontinuation (any reason, virologic failure and toxicity) and each of the samples considered (overall, DTG-containing and suppressed at switch).

In all analyses, time to discontinuation was significantly longer in the TT group. In the DTG-containing sub-analysis, the risk of discontinuation due to any reason was 49% higher in the 2DC group (aHR: 1.49, 95% C.I. [1.22; 1.83]). Risk of virologic failure was also significantly higher in the 2DC group: almost 3-fold higher in the DTG-containing sub-analysis (aHR: 2.78, 95% C.I. [1.71; 4.51]) and almost 2-fold higher (aHR: 1.86, 95% C.I. [1.15; 3.02]) in the HIV RNA<50 copies/mL at baseline sub-analysis. Discontinuation due to adverse events was similar between groups.

## Discussion

In the present study, persistence and probability of remaining free of virologic failure were significantly higher in patients on INSTI-based TT compared to DTG and/or PI/r containing 2DC, with no difference in toxicity. In fact, the present analysis suggests that the risk of virologic failure is at least two times higher with 2DC vs TT when considering both the overall sample and the DTG-containing sub-sample. Perhaps even more relevant is the finding that among patients suppressed at baseline, the risk of virologic failure after controlling for confounders, remained almost twice as high in the 2DC vs TT group. This negative impact in terms of virologic failure associated with 2DC was not compensated by a reduction in discontinuations due to adverse events, thus limiting the evidence supporting a switch to 2DC for toxicity reasons.

TT has been firmly established as an efficacious and effective treatment option over the last 20 years, both in clinical trials [14] and the real-world setting [15, 16]. However, real-world evidence supporting the effectiveness of bPI+3TC, DTG+3TC or DTG+RPV 2DC is limited [17, 18].

The present analysis contributes to the limited body of evidence of real-world effectiveness of contemporary TT and DTG and/or bPI 2DC regimens. To our knowledge this is the first analysis to compare DTG-containing TT to DTG-containing 2DC in the real-world and the results reinforce those of the overall analysis, indicating a significantly higher probability of virologic failure with DTG-containing 2DC with no statistically significant difference in terms of discontinuations due to adverse events. This may seem at odds with the results of the SWORD 1&2 randomised clinical trials, which demonstrated non-inferior efficacy of the combination of DTG plus RPV to that of a previous and on-going standard TT [6]. A likely explanation for this discrepancy relies on the differences in the baseline characteristics between the populations studied in the SWORD 1&2 and in our analysis: participants in the trials were on their first or second line of treatment and were included only if they had not experienced a virological failure and had an excellent virological control in the preceding months. In contrast, the patients in our study were unselected for their past antiretroviral treatment history or virological failure, and were not excluded if the viral load was detectable at the baseline visit. Taken together, both sets of analyses lead to a seemingly coherent hypothesis that, in carefully selected patients (with the characteristics defined as inclusion criteria in the SWORD trials) DTG plus RPV 2DC is as effective as INSTI-based TT, whereas in cases with a less favorable ART background TT provides a significant benefit. It is of concern that the relatively high proportion of patients were switched to a 2DC regimen in suboptimal conditions relative to their ART history. This may be due to the fact that research in so called “less drug regimens” (monotherapy and later 2DC) became very popular in Spain in recent years. Many clinicians became familiar and confident with the use of these oversimplified regimens, probably straining on occasions their indications.

Our analysis has strengths and limitations. Among strengths, the sample size was large, incorporating current TT regimens, including F/TAF-based single-tablet regimens, which, because of their improved safety profile, minimize the toxicity associated with some older TT. The study inclusion criteria specifically excluded 2DC regimens mentioned as not recommended by the European AIDS Society guideline V9.1 (pp. 15 “Strategies not recommended”) [5] and included the largest number to date of patients on 2DC classified as alternatives. Also, patient-regimens that occurred in the framework of a clinical trial were excluded to better reflect the real-world setting. Finally, the observed findings concerning the relationship between current 2DCs and discontinuation occurred in the context of multivariate analysis where, in as much as data were available, differences between groups were controlled for.

The most important limitation of the study is the likelihood of confounding by indication since clinicians select the most adequate treatment for a given patient with far more information than that collected by means of variables in the VACH cohort. It is also unknown if the outcomes of persistence, time to virologic failure, or discontinuations due to adverse events would have been similar to those obtained by patients treated with TT if patients treated with 2DC had been given TT; the aim of the analysis was simply to describe what occurs in clinical practice without an attempt to predict what would have happened in a distinct clinical setting. Patient characteristics were different in the 2DC and TT groups, pointing to a potential worse adherence to treatment of 2DC patients that could impact results despite the efforts to control for confounders. In addition, several covariates of interest were incompletely recorded in the database, precluding their inclusion in the regression analysis. There is an unquestionable heterogeneity in the list of 2DC included in the study. Among a larger list having been used in the Cohort, which was available in the database, we selected those combinations supported by at least one randomised clinical trial, but not necessarily for treatment switches in pre-treated patients (the only exception being the combination of DTG+bDRV). Also, it must be taken into account that at the time of the analyses all 2DC considered were only available as multi-tablet regimens. Last but not least, all available information was used to minimize confounding, namely by including the number of previous virologic failures since ART initiation, number of previous switches for any reason as well as clinical and demographic characteristics. Despite that, the impact of the NRTI backbone, qualitatively specific previous regimens, non-adherence, or other unobservable confounders (not captured in the proxies used) on the results of this study cannot be ruled out.

To provide quality care, minimize adverse events, and improve the lives of patients living with HIV, it is critical to investigate real-world effectiveness of ART regimens. As new ART regimens are developed, great care should be taken to establish real-world efficacy and viral suppression prior to implementation.

## Supporting information

S1 Table Sociodemographic and clinical characteristics at baseline, by sub-analysis and treatment group. TT: triple therapy; 2DC: two-drug combinations; SD: standard deviation; HIV: Human immunodeficiency virus; AIDS: acquired immunodeficiency syndrome; RNA: ribonucleic acid; ART: antiretroviral therapy; RNA; ribonucleic acid.
(DOCX)

S2 Table Patient-regimen end of the observation period status, by sub-analysis and treatment group. TT: triple therapy regimen; 2DC: two-drug combination; HIV: Human Immunodeficiency Virus; RNA; ribonucleic acid.
(DOCX)

S3 Table Final Cox model for two-drug combinations versus triple therapy time to discontinuation, by sub-analysis. Y/N: Yes/No; 2DC: two-drug combination; TT: triple therapy; HIV: Human Immunodeficiency Virus; RNA: ribonucleic acid; aHR: adjusted hazard ratio.
(DOCX)

S4 Table Final Cox model for two-drug combinations versus triple therapy switch due to virologic failure, by sub-analysis. Y/N: Yes/No; 2DC: two-drug combination; TT: triple therapy; HIV: Human Immunodeficiency Virus; RNA: ribonucleic acid; aHR: adjusted hazard ratio.
(DOCX)

S5 Table Final Cox model for two-drug combinations versus triple therapy switch due to toxicity, by sub-analysis. Y/N: Yes/No; 2DC: two-drug combination; TT: triple therapy; HIV: Human Immunodeficiency Virus; RNA: ribonucleic acid; aHR: adjusted hazard ratio.
(DOCX)
