# Supplementary material for: Real world effectiveness of standard of care triple therapy versus two-drug combinations for treatment of people living with HIV
Source: PLoS One. 2021 Apr 8;16(4):e0249515. doi: 10.1371/journal.pone.0249515 (PMC8031389; doi:10.1371/journal.pone.0249515)
Supplement: S1 Table — TT: triple therapy; 2DC: two-drug combinations; SD: standard deviation; HIV: Human immunodeficiency virus; AIDS: acquired immunodeficiency syndrome; RNA: ribonucleic acid; ART: antiretroviral therapy; RNA; ribonucleic acid. (DOCX) [file pone.0249515.s001.docx]

S1 Table: Sociodemographic and Clinical Characteristics at Baseline, by Sub-analysis and Treatment Group.

|  | Dolutegravir-containing  *(N=3,778)* | | |  | *HIV RNA <50 copies/mL at Baseline (N=6,982)* | | |
| --- | --- | --- | --- | --- | --- | --- | --- |
| **Characteristics at Switch** | **TT**  **(N=3,090)** | **2DC**  **(N=688)** | ***P-value*** |  | **TT**  **(N=5,596)** | **2DC**  **(N=1,386)** | ***P-value*** |
| Age, mean (SD) | 48.9 (10.1) | 50.6 (8.7) | **<.0001** |  | 48.0 (10.0) | 50.7 (9.3) | **<.0001** |
| Male, % | 76.6% | 67.6% | **<.0001** |  | 77.1% | 72.8% | **0.001** |
| Prior AIDS diagnosis, % Yes | 25.8% | 32.1% | **0.0008** |  | 23.6% | 29.9% | **<.0001** |
| CD4+ T cell count <350 cells/µL | 22.1% | 27.6% | **0.002** |  | 17.1% | 18.8% | 0.115 |
| HIV RNA <50 copies/mL, % | 76.3% | 73.2% | 0.097 |  | 100% | 100% | 1.000 |
| Illicit drug use, % Yes | 32.7% | 35.0% | 0.244 |  | 32.6% | 40.8% | **<.0001** |
| Hepatitis C virus coinfection, % Yes | 39.8% | 42.7% | 0.187 |  | 39.0% | 47.5% | **<.0001** |
| Hepatitis B virus coinfection, % Yes | 2.2% | 1.7% | 0.413 |  | 4.8% | 1.4% | **<.0001** |
| Years on ART, mean (SD) | 12.5 (7.5) | 15.1 (7.3) | **<.0001** |  | 12.0 (7.4) | 14.8(7.0) | **<.0001** |
| Number of previous ART regimens, mean (SD) | 4.6 (3.7) | 7.8 (5.9) | **<.0001** |  | 4.4 (3.5) | 7.0 (5.0) | **<.0001** |
| Number of virologic failures, mean (SD) | 1.0 (2.0) | 2.6 (4.0) | **<.0001** |  | 0.86 (1.5) | 2.04 (3.0) | **<.0001** |

Legend: TT: triple therapy; 2DC: two-drug combinations; SD: standard deviation; HIV: Human immunodeficiency virus; AIDS: acquired immunodeficiency syndrome; RNA: ribonucleic acid; ART: antiretroviral therapy; RNA; ribonucleic acid.
