# Supplementary material for: Real world effectiveness of standard of care triple therapy versus two-drug combinations for treatment of people living with HIV
Source: PLoS One. 2021 Apr 8;16(4):e0249515. doi: 10.1371/journal.pone.0249515 (PMC8031389; doi:10.1371/journal.pone.0249515)
Supplement: S2 Table — TT: triple therapy regimen; 2DC: two-drug combination; HIV: Human Immunodeficiency Virus; RNA; ribonucleic acid. (DOCX) [file pone.0249515.s002.docx]

S2 Table: Patient-Regimen End of the Observation Period Status, by Sub-analysis and Treatment Group.

|  | Dolutegravir-containing *(N=3,778)* | | |  | *HIV RNA <50 copies/mL at Switch (N=6,982)* | | |
| --- | --- | --- | --- | --- | --- | --- | --- |
| **End of the Observation Period Status, n (%)** | **TT (n=3,090)** | **2DC (n=688)** | ***P-value*** |  | **TT (n=5,596)** | **2DC (n=1,386)** | ***P-value*** |
| Remains on treatment | 2484 (80.4%) | 494 (71.8%) |  |  | 3,910 (69.9%) | 877 (63.3%) |  |
| Reason for change of treatment | 490 (15.9%) | 171 (24.9%) |  |  | 1,248 (22.3%) | 406 (29.3%) |  |
| Virologic failure | 40 (8.2%) | 32 (18.7%) | **<0.001** |  | 47 (3.8%) | 33 (8.1%) | **<0.001** |
| Adverse event | 107 (21.8%) | 20 (11.7%) | **0.004** |  | 273 (21.9%) | 83 (20.4%) | 0.542 |
| Intolerance | 2 (0.4%) | 2 (2.1%) | 0.276 |  | 8 (0.6%) | 3 (0.7%) | 0.737 |
| To avoid long-term toxicity | 18 (3.7%) | 2 (1.2%) | 0.122 |  | 67 (5.4%) | 5 (1.2%) | **<.001** |
| Simplification | 75 (15.3%) | 14 (8.2%) | **0.019** |  | 243 (19.5%) | 72 (17.7%) | 0.439 |
| Drug interactions | 15 (3.1%) | 5 (2.9%) | 1.000 |  | 52 (4.2%) | 18 (4.4%) | 0.817 |
| Loss to follow-up | 8 (1.6%) | 0 (0.0%) | 0.121 |  | 8 (0.6%) | 3 (0.7%) | 0.737 |
| Others | 224 (45.7%) | 96 (56.1%) | **0.019** |  | 545 (43.7%) | 186 (45.8%) | 0.450 |
| *Missing value* | 1 | 0 |  |  | *5* | *3* |  |
| Loss to follow-up | 100 (3.2%) | 17 (2.5%) |  |  | 412 (7.4%) | 97 (7.0%) |  |
| Death | 16 (0.5%) | 6 (0.9%) |  |  | 26 (0.5%) | 6 (0.4%) |  |

Legend: TT: triple therapy regimen; 2DC: two-drug combination; HIV: Human Immunodeficiency Virus; RNA; ribonucleic acid
