# Supplementary material for: Real world effectiveness of standard of care triple therapy versus two-drug combinations for treatment of people living with HIV
Source: PLoS One. 2021 Apr 8;16(4):e0249515. doi: 10.1371/journal.pone.0249515 (PMC8031389; doi:10.1371/journal.pone.0249515)
Supplement: S3 Table — Y/N: Yes/No; 2DC: two-drug combination; TT: triple therapy; HIV: Human Immunodeficiency Virus; RNA: ribonucleic acid; aHR: adjusted hazard ratio. (DOCX) [file pone.0249515.s003.docx]

S3 Table: Final Cox Model for Two-Drug Combinations versus Triple Therapy Time to Discontinuation, by Sub-analysis.

|  |  | Dolutegravir-containing | |  | *HIV RNA <50 copies/mL at baseline* | |
| --- | --- | --- | --- | --- | --- | --- |
| **Variable at Switch** |  | **aHR [95% C.I.]** | ***P-value*** |  | **aHR [95% C.I.]** | ***P-value*** |
| Therapy group (2DC vs TT) |  | 1.49 [1.22, 1.83] | **<.0001** |  | 1.23 [1.08, 1.40] | **0.003** |
| Hepatitis C virus coinfection (Y/N) |  | 1.51 [1.26, 1.81] | **<.0001** |  | 1.16 [0.98, 1.38] | 0.08 |
| Hepatitis B virus coinfection (Y/N) |  | 1.69 [1.05, 2.70] | **0.030** |  | 1.33 [1.04, 1.71] | **0.03** |
| Years on antiretroviral therapy |  | 0.95 [0.93, 0.96] | **<.0001** |  | 0.95 [0.94, 0.96] | **<.0001** |
| Number of previous regimens |  | 1.09 [1.07, 1.11] | **<.0001** |  | 1.07 [1.05, 1.09] | **<.0001** |
| HIV RNA (≥50 vs. < 50 copies/mL) |  | 1.58 [1.32, 1.89] | **<.0001** |  |  |  |
| Age, per year older |  |  |  |  | 1.01 [0.99, 1.01] | 0.08 |
| Illicit drug use (Y/N) |  |  |  |  | 1.17 [0.99, 1.49] | 0.07 |
| Male (Y/N) |  |  |  |  | 0.89 [0.78, 1.01] | 0.06 |

Legend: Y/N: Yes/No; 2DC: two-drug combination; TT: triple therapy; HIV: Human Immunodeficiency Virus; RNA: ribonucleic acid; aHR: adjusted hazard ratio
