# Supplementary material for: Real world effectiveness of standard of care triple therapy versus two-drug combinations for treatment of people living with HIV
Source: PLoS One. 2021 Apr 8;16(4):e0249515. doi: 10.1371/journal.pone.0249515 (PMC8031389; doi:10.1371/journal.pone.0249515)
Supplement: S4 Table — Y/N: Yes/No; 2DC: two-drug combination; TT: triple therapy; HIV: Human Immunodeficiency Virus; RNA: ribonucleic acid; aHR: adjusted hazard ratio. (DOCX) [file pone.0249515.s004.docx]

S4 Table: Final Cox Model for Two-Drug Combinations versus Triple Therapy Switch Due to Virologic Failure, by Sub-analysis.

|  |  | Dolutegravir-containing | |  | *HIV RNA <50 copies/mL at baseline* | |
| --- | --- | --- | --- | --- | --- | --- |
| **Variable at Switch** |  | **aHR [95% C.I.]** | **P-value** |  | **aHR [95% C.I.]** | **P-value** |
| Therapy group (2DC vs TT) |  | 2.78 [1.71, 4.51] | **<.0001** |  | 1.86 [1.15, 3.02] | **0.01** |
| Number of previous virologic failures |  | 1.12 [1.09, 1.15] | **<.0001** |  | 1.11 [1.01;1.21] | **0.02** |
| HIV RNA (≥50 vs. < 50 copies/mL) |  | 4.78 [2.94, 7.76] | **<.0001** |  |  |  |
| Illicit drug use (Y/N) |  |  |  |  | 2.14 [1.35;3.39] | **0.001** |
| Number of previous regimens |  |  |  |  | 1.04 [0.98, 1.11] | 0.22 |

Legend: Y/N: Yes/No; 2DC: two-drug combination; TT: triple therapy; HIV: Human Immunodeficiency Virus; RNA: ribonucleic acid; aHR: adjusted hazard ratio
