# Supplementary material for: Real world effectiveness of standard of care triple therapy versus two-drug combinations for treatment of people living with HIV
Source: PLoS One. 2021 Apr 8;16(4):e0249515. doi: 10.1371/journal.pone.0249515 (PMC8031389; doi:10.1371/journal.pone.0249515)
Supplement: S5 Table — Y/N: Yes/No; 2DC: two-drug combination; TT: triple therapy; HIV: Human Immunodeficiency Virus; RNA: ribonucleic acid; aHR: adjusted hazard ratio. (DOCX) [file pone.0249515.s005.docx]

S5 Table: Final Cox Model for Two-Drug Combinations versus Triple Therapy Switch Due to Toxicity, by Sub-analysis.

|  |  | Dolutegravir-containing | |  | HIV RNA <50 copies/mL at baseline | |
| --- | --- | --- | --- | --- | --- | --- |
| **Variable at Switch** |  | **HR [95% C.I.]** | ***P-value*** |  | **HR [95% C.I.]** | ***P-value*** |
| Therapy group (2DC vs TT) |  | 1.00 [0.63, 1.58] | 0.99 |  | 1.15 [0.89, 1.49] | 0.29 |
| Age, per year older |  | 1.01 [0.99, 1.03] | 0.22 |  | 1.01 [1.00, 1.03] | **0.005** |
| Years on antiretroviral therapy |  | 0.90 [0.87, 0.93] | **<.0001** |  | 0.89 [0.87, 0.92] | **<.0001** |
| Number of previous regimens |  | 1.07 [1.01, 1.13] | **0.02** |  | 1.13 [1.07, 1.18] | **<.0001** |
| Hepatitis B virus coinfection (Y/N) |  |  |  |  | 1.52 [0.99, 2.32] | 0.05 |
| Number of previous virologic failures |  |  |  |  | 0.90 [0.83, 0.98] | **0.01** |

Legend: Y/N: Yes/No; 2DC: two-drug combination; TT: triple therapy; HIV: Human Immunodeficiency Virus; RNA: ribonucleic acid; aHR: adjusted hazard ratio
